# Comparison of Global and Regional Compliance-Guided Positive End-Expiratory Pressure Titration on Regional Lung Ventilation in Moderate-to-Severe Pediatric Acute Respiratory Distress Syndrome

**DOI:** 10.3389/fmed.2022.805680

**Published:** 2022-05-23

**Authors:** Hong Ren, Li Xie, Zhulin Wang, Xiaoliao Tang, Botao Ning, Teng Teng, Juan Qian, Ying Wang, Lijun Fu, Zhanqi Zhao, Long Xiang

**Affiliations:** ^1^Department of Critical Care Medicine, Shanghai Children’s Medical Center, School of Medicine, Shanghai Jiao Tong University, Shanghai, China; ^2^Clinical Research Institute, School of Medicine, Shanghai Jiao Tong University, Shanghai, China; ^3^Department of Cardiology, Shanghai Children’s Medical Center, School of Medicine, Shanghai Jiao Tong University, Shanghai, China; ^4^Department of Biomedical Engineering, Fourth Military Medical University, Xi’an, China; ^5^Institute of Technical Medicine, Furtwangen University, Villingen-Schwenningen, Germany; ^6^Department of Neonatal, Second People’s Hospital of Kashgar, Xinjiang, China

**Keywords:** acute respiratory distress syndrome, lung protective ventilation, electrical impedance tomography, pediatrics, respiratory compliance

## Abstract

**Purpose:**

To investigate the difference in the positive end-expiratory pressure (PEEP) selected with chest electrical impedance tomography (EIT) and with global dynamic respiratory system compliance (C_rs_) in moderate-to-severe pediatric acute respiratory distress syndrome (pARDS).

**Methods:**

Patients with moderate-to-severe pARDS (PaO_2_/FiO_2_ < 200 mmHg) were retrospectively included. On the day of pARDS diagnosis, two PEEP levels were determined during the decremental PEEP titration for each individual using the best compliance (PEEP_C_) and EIT-based regional compliance (PEEP_EIT_) methods. The differences of global and regional compliance (for both gravity-dependent and non-dependent regions) under the two PEEP conditions were compared. In addition, the EIT-based global inhomogeneity index (GI), the center of ventilation (CoV), and standard deviation of regional delayed ventilation (RVD_SD_) were also calculated and compared.

**Results:**

A total of 12 children with pARDS (5 with severe and 7 with moderate pARDS) were included. PEEP_C_ and PEEP_EIT_ were identical in 6 patients. In others, the differences were only ± 2 cm H_2_O (one PEEP step). There were no statistical differences in global compliance at PEEP_C_ and PEEP_EIT_ [28.7 (2.84–33.15) vs. 29.74 (2.84–33.47) ml/cm H_2_O median (IQR), *p* = 0.028 (the significant level after adjusted for multiple comparison was 0.017)]. Furthermore, no differences were found in regional compliances and other EIT-based parameters measuring spatial and temporal ventilation distributions.

**Conclusion:**

Although EIT provided information on ventilation distribution, PEEP selected with the best C_rs_ might be non-inferior to EIT-guided regional ventilation in moderate-to-severe pARDS. Further study with a large sample size is required to confirm the finding.

## Introduction

Pediatric acute respiratory distress syndrome (pARDS) was proposed by the 2015 Pediatric Acute Lung Injury Consensus Conference (PALICC) (1). The multinational survey data of the pediatric acute lung injury and sepsis investigators (PALISI) have shown that the mortality of pARDS is about 22–40% and the mortality related to pARDS can reach more than 30% in a pediatric intensive care unit (PICU) (2).

Similar to adult ARDS, the pathophysiological characteristics of pARDS include the heterogeneity of lung lesions and the formation of regional collapse in gravity-dependent areas. At present, mechanical ventilation is still the main life-supporting method for the treatment of ARDS. During mechanical ventilation, unreasonable positive end-expiratory pressure (PEEP) setting could cause excessive alveolar expansion and periodic collapse–opening and release of inflammatory mediators, resulting in ventilator-induced lung injury (VILI). It may also lead to hemodynamic instability secondary to right ventricular dysfunction (3, 4). PALICC recommends lung recruitment and titration of the optimal PEEP in pARDS (1). The optimal PEEP keeps alveolar from derecruitment in gravity-dependent areas and introduces only limited alveolar overdistension in non-gravity-dependent areas. In pARDS, the question of how to titrate PEEP is still under debate.

Electrical impedance tomography (EIT) is an advanced non-invasive ventilation monitoring technology at the bedside (5, 6). It can directly visualize whether the collapsed lung areas can be opened after the lung recruitment maneuver, guide the ventilator parameter settings, and minimize VILI. Several recent reviews have summarized the applications of EIT in the treatment of adult ARDS (7, 8). Two randomized controlled studies indicated that EIT-guided PEEP titration may reduce ARDS mortality (9, 10). In terms of children, Rosemeier et al. reported that during lung recruitment and PEEP titration, EIT could help to minimize regional alveolar overdistension and collapse and improve oxygenation (11). However, EIT is not widely used in PICU. We hypothesized that the traditional PEEP titration method using dynamic respiratory system compliance (C_rs_) could lead to similar PEEP and regional ventilation for lung protection in pARDS.

## Materials and Methods

### Study Design

This study was retrospective observational and was conducted in the PICU of Shanghai Children’s Medical Center from 1 January 2020 to 31 December 2020. The Ethics Committee of Shanghai Children’s Medical Center approved this study (SCMCIRB-Y20200087). For all of the included children, written informed consent was obtained from their legal guardians.

### Patients

Children with moderate and severe pARDS diagnosed in accordance with the 2015 PALICC (1) and treated with invasive endotracheal intubation and lung protective ventilation were included. The exclusion criteria were as follows: (1) age > 18 years or weight < 7 kg; (2) uncorrected hemodynamic instability or decompensated shock; (3) serious lesions and trauma of the thorax and spine, surgical incision, and lesions of the relevant parts of the skin, serious deformity of the thorax, and inability to fix EIT bandage; (4) severe obesity and Body Mass Index > 50; (5) implanted intrathoracic devices, such as a cardiac pacemaker, cardiac defibrillator, or any other surgical implant; (6) ongoing cardiac defibrillation; (7) congenital heart disease, congenital diaphragmatic hernia, and severe airway obstructive diseases; (8) sildenafil or inhaled nitric oxide used to treat pulmonary hypertension for various reasons; (9) severe craniocerebral injury, intracranial pressure monitoring, or extraventricular drainage; and (10) high-frequency ventilation.

All of the children continued to receive midazolam, fentanyl sedation, analgesia, and rocuronium neuromuscular block, without spontaneous breathing. The choice of drugs, dosage, and time of administration was determined by PICU attending physicians. All of the patients were treated with low tidal volume lung protection ventilation in the supine position. Pressure control mode was adopted for mechanical ventilation, with a targeted tidal volume of 4–6 ml/predicted body weight, and FiO_2_ was titrated to obtain peripheral blood oxygen saturation between 92 and 100%.

### Electrical Impedance Tomography Monitoring

Electrical impedance tomography measurement was performed to monitor ventilation distribution at the bedside (Pulmovita 500, Dräger Medical, Lübeck, Germany). A belt with sixteen electrodes was placed on the transverse section around the patient’s chest. A reference electrode was placed on the abdomen. The EIT image was continuously recorded and stored at 20 Hz. The respiratory data of the ventilator were transmitted to the EIT device and recorded through the MEDIBUS connection. The tidal ventilation image was divided into ventral and dorsal regions.

### Positive End-Expiratory Pressure Titration Procedures

The positive end-expiratory pressure titration was performed within 6 h of pARDS diagnosis. No lung recruitment maneuver was performed before the PEEP titration. The initial PEEP was set at 15 cm H_2_O. The PEEP was reduced by a step of 2 cm H_2_O and a length of 2 min until the PEEP reached 5 cm H_2_O.

**FIGURE 1:**
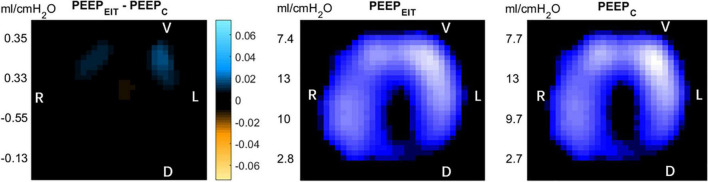
Left: After the positive end-expiratory pressure (PEEP) titration by the two methods, the difference of electrical impedance is displayed on the electrical impedance tomography (EIT) diagram. Blue: compliance gain; Orange: compliance loss. Middle: PEEP titrated according to the regional compliance method. Right: PEEP titrated according to global dynamic respiratory system compliance. R, right; L, left V, Ventral D, dorsal.

“Optimal” PEEP was determined with EIT (PEEP_EIT_) and C_rs_ (PEEP_C_). PEEP_EIT_ was selected to minimize regional overdistention and collapsed based on regional compliance (12). PEEP_C_ was selected when C_rs_ reached its maximum during the decremental PEEP trial (13) (Figure 1).

Tidal relative impedance changes were normalized to tidal volume in milliliters. Regional C_rs_ for ventral and dorsal regions were calculated as tidal impedance changes in ventral and dorsal regions over driving pressure, respectively. Several EIT-based parameters were calculated to assess the spatial and temporal regional ventilation distribution. They were global inhomogeneity (GI) index (14), center of ventilation (CoV), (15) and the standard deviation of regional ventilation delayed ventilation (RVD_SD_) (16).

Data reconstruction was conducted using the Draeger reconstruction method *via* Draeger EIT Data Analysis Tool (version 63, Dräger Medical, Lübeck, Germany), and the data were analyzed using a customized software using Matlab (The Math-Works, Natick, MA, United States).

### Statistical Analysis

The data complying with normal distribution were presented as mean ± SD. The monitoring parameters between the two methods were compared with a paired-sample *t*-test. Non-normally distributed data were represented by median (interquartile range) and were compared with Wilcoxon signed-rank test. SPSS 24.0 software package (SPSS, Chicago, Illinois, United States) and MedCalc 11.4.3.0 software (Mariakel, Belgium) were used for statistical analysis. The *p-*values lower than 0.05 were considered to be statistically significant. The Holm-Bonferroni method was used to adjust the significant levels for multiple comparisons.

## Results

**FIGURE 2:**
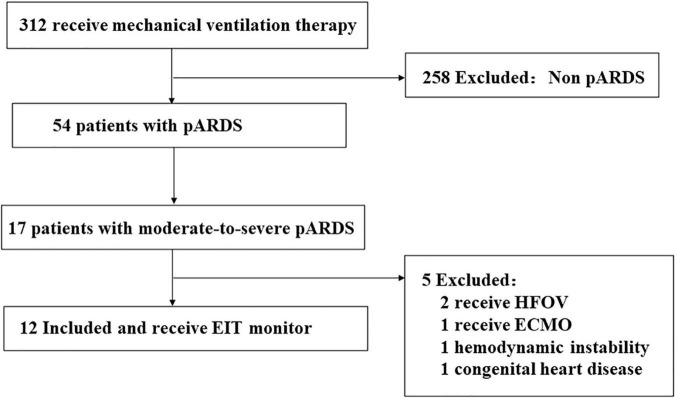
Flowchart of patient enrollment in this study. HFOV, High frequency oscillation ventilation; ECMO, Extracorporeal membrane oxygenation.

In 2020, there were 312 pediatric patients ventilated in our department. Among them, only 54 patients were pARDS and only 17 were moderate-to-severe. We illustrated the patient enrollment process with the following flowchart (Figure 2).

We included a total of 12 children with pARDS, including 5 with severe pARDS and 7 with moderate pARDS. The clinical characteristics of the 12 children are shown in Table 1.

**TABLE 1:** The clinical characteristics of 12 included children with Pards.

Patients	Age (month)	Gender	Weight (kg)	PaO_2_/FiO_2_	FiO_2_	PEEP	Sputum etiology	Blood etiology	Underling diseases	Outcome
1	16	Female	10	72.33	0.6	7	*Enterobacter aerogenes*	Methicillin-resistant staphylococcus	Post liver transplantation	Survived
2	36	Female	17	76.22	0.9	9	Parainfluenza virus *Streptococcus pneumoniae*	Human staphylococcus	Acute lymphoblastic leukemia	Survived
3	108	Female	41	88.42	0.95	13	None	*Staphylococcus epidermidis*	Acute lymphoblastic leukemia	Deceased
4	35	Male	13	88.43	0.7	9	Parainfluenza virus	Epstein-Barr virus	Lymphoma	Survived
5	13	Male	9	91.25	0.8	9	*None*	None	High IgM immunodeficiency disease	Deceased
6	5	Male	8.5	125.8	0.5	6	*Candida albicans*	None	Rhabdomyosarcoma	Survived
7	48	Male	18.3	141.83	0.6	9	*Escherichia coli* (CRE)	None	Acute lymphoblastic leukemia	Survived
8	65	Male	20	143.33	0.6	10	*Pneumocystis carinii*	None	Acute lymphoblastic leukemia	Survived
9	5	Male	8	143.33	0.6	6	*Acinetobacter baumannii, Klebsiella pneumoniae*	None	Post liver transplantation	Survived
10	84	Male	14.5	168.33	0.6	8	*Pneumocystis carinii*	None	Congenital hypogammaglobulinemia	Survived
11	144	Male	40	175.11	0.6	9	*Pneumocystis carinii*	None	Acute lymphoblastic leukemia	Survived
12	72	Female	23	181.67	0.6	7	*Mycoplasma pneumoniae*	None	None	Survived

There was no differences in global compliance at PEEP_C_ and that at PEEP_EIT_ [28.7 (2.84–33.15) vs. 29.74 (2.84–33.47)] ml/cm H_2_O, *p* = 0.028 (the Holm-Bonferroni method adjusted significant level was 0.017). No differences were found in non-dependent area (*p* = 0.028 adjusted level 0.025) and gravity-dependent area (*p* = 0.207) (Table 2).

**TABLE 2:** The comparison between the global compliance and regional compliance at PEEP_EIT_ and PEEP_C_.

	OD/CL	Global dynamic respiratory system compliance	*P*
PEEP	9.83 ± 2.17	10.05 ± 2.43	0.104
Global compliance _T_	28.7 (2.84–33.15)	29.74 (2.84–33.47)	0.028^#^
Non-gravity-dependent area compliance	12.2 (1.34–17.11)	12.46 (1.34–18.41)	0.028^##^
Gravity-dependent area compliance	13.82 (1.58–17.11)	14.93 (1.58–18.28)	0.207

*^#^The Holm-Bonferroni method adjusted significant level was 0.017.*

*^##^The Holm-Bonferroni method adjusted significant level was 0.025.*

**FIGURE 3:**
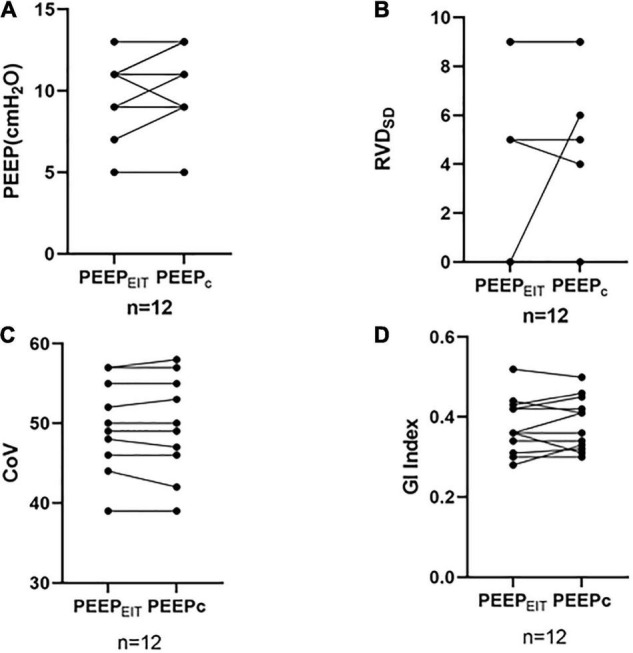
**(A)** No differences were found in the PEEP between PEEP_EIT_ and PEEP_C_. **(B)** No differences were found in the standard deviation of regional delayed ventilation (RVD_SD_) between PEEP_EIT_ and PEEP_C_. **(C)** No differences were found in CoV between PEEP_EIT_ and PEEP_C_. and **(D)** No differences were found in GI Index between PEEP_EIT_ and PEEP_C_.

No differences were found in RVD_SD,_ GI, and CoV between PEEP_EIT_ and PEEP_C_ (RVD_SD_: 2.33 ± 3.65 vs. 2.75 ± 3.67, *p* = 0.435; GI: 0.378 ± 0.693 vs. 0.384 ± 0.663, *p* = 0.522; CoV: 48.75 ± 6.09 vs. 48.67 ± 6.45, *p* = 0.723) (Figure 3).

## Discussion

In the present study, we compared the PEEP selected with maximum global C_rs_ and the EIT-based regional C_rs_ methods in pARDS. The PEEP levels selected with these two methods did not differ. Both global and regional C_rs_ and spatial and temporal ventilation distribution were comparable. PEEP selected with best C_rs_ might be non-inferior to EIT-guided one with respect to regional ventilation. When regional C_rs_ is unavailable, global C_rs_ could be a practical surrogate to guide PEEP titration.

The traditional method of titrating PEEP is based on the parameters of global parameters, including P-V curve inflection points in the P-V loop and optimal results of blood gas analysis (PaO_2_, PaCO_2_, and dead space fraction) (13, 17). The computed tomography scanning method could provide regional information (18) but it is impractical due to the need for transportation and radiation exposure. PEEP titration with transesophageal pulmonary pressure titration could be promising. However, in a study of 200 patients with moderate-to-severe ARDS, Beitler et al. (19) found that, when compared with the empirical PEEP-FiO_2_ method, the optimal PEEP by transesophageal pulmonary pressure titration did not improve the prognosis (*p* = 0.88) and mechanical ventilation time (*p* = 0.85). This study showed that due to the heterogeneity of ARDS lesions, the parameters reflecting only global lung ventilation may not represent the regional lung lesions. The optimal PEEP value is different not only before and after lung injury but also between dorsal and ventral regions. Therefore, it is necessary to monitor the compliance of different regions. EIT provides information on regional ventilation at the bedside. Costa et al. (12) and Meier et al. (20) reported that EIT can be used to monitor regional compliance. Lowhagen et al. (21) proposed a so-called “intra tidal gas distribution,” which divides regional tidal impact into eight isovolume parts, to identify tidal changes and overdistension in the lung regions. Zhao et al. (22) reported better compliance value and oxygenation index (PaO_2_/FiO_2_) in patients with ARDS in whom PEEP was titrated according to the OD/CL method than those titrated with PEEP using P-V curve. They also concluded that if the study population doubled and the intergroup mortality remained unchanged, the mortality of the PEEP group titrated according to the regional compliance would be significantly lower. Recently, Hsu et al. (9) and He et al. (10) reported that PEEP titrated by the regional C_rs_ method can reduce mortality when compared with traditional methods in adult patients with ARDS.

Although pARDS and adult ARDS share the same pathological and pathophysiological characteristics, the data of adult ARDS clinical studies are not always applicable to the pARDS. The pediatric and adult patients are different at least in the following aspects: (1)the definition of pARDS in the PALICC is different from adults ARDS in Berlin definition:(a) the definition of pARDS eliminates the requirement of bilateral infiltration in chest imaging; (b) use oxygen index (OI) and Oxygen saturation index (OSI) instead of PaO_2_/FiO_2_ ratio with minimum PEEP level; (c) pARDS is defined for special children with chronic lung disease or cyanotic congenital heart disease (23). (2) Compared with adults ARDS, the definition pARDS have lower requirements for respiratory mechanical parameters. Taking compliance as an example, the elasticity of the chest wall in children is significantly reduced, and the elastin and collagen components of the lung change with age (24). In addition, the respiratory system compliance cannot be accurately calculated because the tidal volume delivered to lungs is not accurately measured in children:(a) there is serious air leakage around the endotracheal tube, which may be minimized in patients with severe pARDS because of the cuffed endotracheal tubes; (b) the ideal weight measurement of children is more complex, especially those with severe scoliosis; (c) the device and location of the device (proximal airway vs. at the ventilator) to measure tidal volume may result in different values for tidal volume, based on the type of ventilator, circuit tubing used, compliance of the patient, compliance of the tubing, and size of the patient. For these reasons, PALICC does not recommend the use of compliance in the definition of pARDS. (3) There are few prospective studies on the optimal PEEP level of pARDS, resulting in no unified recommended method for PEEP titration in the treatment of pARDS with mechanical ventilation. The risk of high PEEP may be related to the adverse results of cardiopulmonary interaction caused by low chest wall elasticity in children or newborns (25, 26). Khemani et al. (27) reported that the PEEP setting lower than the recommended PEEP setting of ARDSNet will increase the mortality of pARDS. The study from Khemani et al. confirmed that PEEP was independently related to the mortality of pARDS, and clearly pointed out the necessity of prospective study on optimizing the PEEP in pARDS.

Due to the uniqueness of children and the limitations of monitoring tools, the above-mentioned findings in adult ARDS might not be directly applicable to pARDS. EIT pediatric electrodes are still in development. Since global C_rs_ is monitored throughout the entire period of mechanical ventilation, it could be used for PEEP titration in the absence of EIT.

It should be noted that the global dynamic respiratory compliance used in our study could be different from the static compliance measured during volume controlled and with inspiratory hold. Stahl et al. (28) suggested that the application of dynamic respiratory mechanics as a diagnostic tool in ventilated patients should be more appropriate than using static lung mechanics.

Several studies have reported that there is a correlation between the OD/CL method and the global respiratory system compliance method. Su et al. (29) showed in 18 patients with ARDS that during the decline of PEEP, PEEP titrated by the OD/CL method correlated with PEEP selected for the best compliance of the respiratory system. In an animal study, Bikker et al. (30) found a correlation between the optimal PEEP based on the EIT regional compliance and PEEP titrated according to the global compliance in eight porcine ALI models induced by oleic acid. In addition, Puel et al. (31) reported that in patients with severe ARDS undergoing venous-venous extracorporeal membrane oxygenation (V-V ECMO), the OD/CL was consistent with PEEP compliance (PEEPcomp) titrated according to global compliance. However, based on the fact that OD is more harmful to patients than CL, some researchers revised the OD/CL balance scheme and proposed “OD/CL15” that allows 15% CL. The ratio definition minimizes the alveolar OD (32, 33). OD/CL15 leads to the reduction of the optimal PEEP and increases the risk of lung collapse. Franchineau et al. (34) examined 15 patients with ARDS and found that the optimal PEEP set according to OD/CL15 was different from the PEEP estimated by the optimal compliance of the lung. It showed that the PEEP titration based on EIT considered not only the global compliance but also the balance between overdistension and collapse in different regions, whereas the PEEP titrated according to the global optimal compliance may not represent the optimal ventilator ventilation parameters.

In the present study, we evaluated three EIT-based parameters to assess the spatial and regional ventilation distributions, namely the GI, CoV, and RVD_SD_ indices. Frerichs et al. (35) reported that in neonatal respiratory distress syndrome (NRDS) the use of pulmonary surfactant can transfer the CoV of children from a non-gravity-dependent area to a gravity-dependent area. Another study showed a significant difference between PEEP measured according to GI and the value recommended by ARDSnet guidelines, and PEEP value did not correlate with PaO_2_/FiO_2_ (36). RVD may reveal the degree of tidal recruitment if the PEEP is inadequate (16). It may also be used to evaluate the diaphragm activities during spontaneous breathing tests (37).

In the present study, no significant differences in CoV, GI, or RVD_SD_ between PEEP_EIT_ and PEEP_C_ were found, which indicates that the ventilation heterogeneity of pARDS may not be as large as that of adult ARDS.

This study has several limitations. First, the very small sample size limited the conclusions. The huge variation in age (from 5 to 144 months) and therefore body height might be another major limitation of this study. Karsten et al. (38) showed that the clinical availability and rationality of EIT measurement depend on appropriate belt position, impedance visualization, correct analysis, and data interpretation. When EIT is used to estimate global parameters, such as tidal volume or end expiratory lung volume changes, the optimal electrode plane is between the 4th and 5th intercostal spaces. In our study, due to the various age and weights of children, the EIT belt was uniformly placed at the connecting line at the nipple level. Brabant et al. (39) show that the volume-impedance ratio may vary depending on the PEEP levels. To ensure that the belt position was adequate and PEEP-dependent volume-impedance ratio did not significantly influence the results, we first evaluated whether the volume-impedance ratios at different PEEP were significantly different. With the adequate belt positioning, no difference in volume-impedance ratios at different PEEP levels was found. Then, the mean ratio of volume-impedance at all PEEP was used. Bikker et al. (40) showed that induced cranio-caudal shift of lung tissue may alter the proportion of the lung which is captured within the EIT sensitivity region in adult patients with ARDS. In our study, this effect of PEEP-induced cranio-caudal shifting may be even more pronounced in patients with smaller lungs (e.g., 4 months) compared to older/taller children. The small sample size and the huge variation in lung size in this cohort may have led to the non-significant result. According to the current research results, future clinical trials should focus on increasing the number of cases with pARDS, exploring the EIT on the global and regional ventilation of pARDS, and guiding the implementation of pulmonary protective ventilation strategy of pARDS.

## Conclusion

During lung protection ventilation therapy in moderate-to-severe pARDS, although EIT provided information of ventilation distribution, PEEP selected with best C_rs_ might be non-inferior to EIT-guided one regarding regional ventilation in moderate-to-severe pARDS. EIT can monitor lung regional compliance and enrich the understanding of pARDS lung protection ventilation by monitoring GI, CoV, RVD, and other parameters. More patients should be included in future larger, possibly multicenter, clinical trials to explore the clinical efficacy of EIT for lung protective ventilation in pARDS.

## Data Availability Statement

The original contributions presented in the study are included in the article/supplementary material, further inquiries can be directed to the corresponding author/s.

## Ethics Statement

The studies involving human participants were reviewed and approved by the Ethics Committee of Shanghai Children’s Medical Center (SCMCIRB-Y20200087). Written informed consent to participate in this study was provided by the participants’ legal guardian/next of kin. Written informed consent was obtained from the minor(s)’ legal guardian/next of kin for the publication of any potentially identifiable images or data included in this article.

## Author Contributions

LoX, LF, ZZ, HR, and LiX were responsible for the literature search, study design, writing, and critical revision. ZW, XT, BN, TT, JQ, and YW mainly participated in data collection, data analysis, and data interpretation. All authors have read and approved the final manuscript.

## Conflict of Interest

The authors declare that the research was conducted in the absence of any commercial or financial relationships that could be construed as a potential conflict of interest.

## Publisher’s Note

All claims expressed in this article are solely those of the authors and do not necessarily represent those of their affiliated organizations, or those of the publisher, the editors and the reviewers. Any product that may be evaluated in this article, or claim that may be made by its manufacturer, is not guaranteed or endorsed by the publisher.
